# Research on the impact of the ESG rating divergence of manufacturing firms on carbon emission intensity

**DOI:** 10.1371/journal.pone.0323929

**Published:** 2025-06-02

**Authors:** Jialu Deng, Wenhe Lin, Jiuzhen Huang, Yunying Cai, Wenbao Wang

**Affiliations:** 1 College of Economics and Management, Fujian Agriculture and Forestry University, Fuzhou, China; 2 College of Construction Engineering, Fuzhou Polytechnic, Fuzhou, China; South China Normal University School of Economics and Management, CHINA

## Abstract

The task of reducing carbon emission intensity is difficult for manufacturing firms in China under the national goal of “carbon peaking and carbon neutrality”. Numerous studies have confirmed the inhibitory impact of ESG performance on carbon emission intensity; nevertheless, the significance of ESG rating divergence as a derivative of ESG ratings has been neglected. Therefore, this study selects Chinese A-share listed manufacturing firms from 2011–2022 as a research sample and empirically examines the impact of ESG rating divergence on the carbon emission intensity of Chinese manufacturing firms via a higher-order fixed effects model. The study revealed that (1) ESG rating divergence creates disincentives for manufacturing firms to increase carbon emission intensity; (2) ESG rating divergence leads to an increase in corporate carbon emissions by inhibiting incentives for green innovation; and (3) mitigating financial constraints and enhancing digital transformation in enterprises diminishes the impact of ESG rating divergence on the carbon emissions of manufacturing firms. Furthermore, enterprise digital transformation can exert a moderating influence on both the initial and subsequent stages of the “ESG performance→green innovation→carbon emission intensity” pathway, predominantly in the initial phase. (4) The impact of ESG rating divergence on carbon emission intensity varies depending on the ownership, industry, geography, and level of competitiveness of manufacturing enterprises. The findings not only provide empirical evidence on the feasibility of expanding the standardization of ESG ratings within China’s regulatory framework but also provide useful inspiration for Chinese firms to reduce their carbon emission intensity and achieve sustainable development.

## Introduction

According to the Global Carbon Budget 2024 report released by the GCP (Global Carbon Project), anthropogenic carbon dioxide emissions will reach a record 41.6 billion tons in 2024, 2% increase from 2023 and China’s carbon dioxide emissions are expected to account for 32% of the world’s total, ranking first in the world. China, being the largest carbon emitter globally and classified as a developing country, established a goal in 2020 to ‘reach the peak of CO2 emissions before 2030 and attain carbon neutrality before 2060’ [1], namely, carbon peaking and carbon neutrality goals. Realizing this goal means reducing carbon emission intensity. Enterprises are the main drivers of production processes and carbon emissions, and the manufacturing industry is the main contributor to China’s industrial energy consumption, which means that focusing on the carbon emissions of manufacturing enterprises is crucial to the implementation of the carbon peaking and carbon neutrality goals.

The United Nations Environment Programme established in 2004, an investment theory and metric for valuing companies that focuses on the level of green development, modern governance and social responsibility of companies, is known as ESG. It measures how a company contributes to sustainable development [2,3]. A significant portion of the current literature investigates the role of ESG performance in facilitating carbon emission reductions [4–6]. As ESG practices evolve, certain scholars are examining the issues associated with ESG ratings, including insufficient transparency [7], ambiguous criteria [8], inconsistency [9], and the profit motives of rating agencies [10]. These factors lead to notable discrepancies in the ESG ratings of the same publicly listed company as those assessed by various rating agencies. Research indicates that the correlation between ratings from different agencies ranges from approximately 0.38–0.71 [11,12]. The impact of ESG rating divergence is widely perceived as detrimental, as such divergence can disseminate misleading information to market participants, which may not only adversely influence corporate market returns and decision-making processes but also exacerbate information asymmetry between investors and listed companies [10,13], reduce the importance of corporate environmental governance or even foster skepticism regarding the sustainability of firms [14].

Although the literature has verified that ESG ratings can reduce carbon emissions, at the national and corporate levels [15–17], research on the effect and mechanism of ESG rating divergence, as ESG rating-derived information, on the carbon emission intensity of corporations has not yet been carried out in large quantities. Therefore, this study aims to explore whether ESG rating divergence affects manufacturing companies’ ability to balance emission reduction and development. What are the mechanisms? Do differences in external factors produce different effects? The answers to these questions not only provide empirical evidence on the feasibility of expanding the standardization of ESG ratings within China’s regulatory framework but also provide useful insights into how ESG ratings can be better utilized to contribute to the achievement of the carbon peaking and carbon neutrality goals at the national level. On the basis of the above considerations, this study uses 2011–2022 China A-share manufacturing companies as the research sample to empirically test the relationship between ESG rating divergence and carbon emission intensity, contributing to the understanding of the environmental impacts of ESG rating divergence among Chinese manufacturing firms. Second, this study emphasizes the value of green innovations in improving ESG rating divergence and carbon emissions in manufacturing enterprises at both the theoretical and empirical levels. Furthermore, we analyze and explore the mechanisms through which financing constraints and the degree of digital transformation influence the varying carbon emissions of manufacturing firms’ ESG ratings. Finally, this study performs a regression analysis of Chinese manufacturing enterprises, considering ownership, area, industry, and degree of market competition. The aim is to more accurately quantify the diverse impacts of carbon emissions that arise from the ESG rating divergence of these manufacturing firms.

## Theoretical analysis and hypotheses formulation

### ESG rating divergence and carbon emission intensity

ESG ratings supplement conventional financial disclosure and constitute a significant component of nonfinancial disclosure [18]. According to signaling theory, corporations can deliberately convey signals to external entities to mitigate information asymmetry and fulfill their aims [19]. ESG disclosure is highly regarded due to its widespread acceptance among various market participants [20]. The benefits of CSR investments extend beyond improved decision-making for stakeholders [21]. They also demonstrate the government’s and financial institutions’ acknowledgment of these investments and enhance favorable firm reputation inside the capital market [22]. ESG ratings are essential in linking firms with the market, providing effective market incentives for firms aiming to lower carbon emissions [23]. Every aspect of ESG performance, including environmental, social, and governance factors, clearly plays a significant role in encouraging long-term success and lowering corporate carbon emissions [15]. Numerous empirical studies have shown the close correlation between a corporation’s environmental performance, particularly in relation to energy consumption, energy security, and food security, and its greenhouse gas emissions [24]. The environmental aspect of ESG performance, in contrast, centers on ways in which businesses may reduce carbon emissions through measures such as renewable energy, trash reduction, and efficient use of resources. Nonetheless, the issues of insufficient transparency, ambiguous criteria, inconsistency, and profit-driven motives of rating agencies may compel certain companies to emit “hypocritical” signals externally, such as inflating their ESG ratings [7–10].

From an internal management perspective, the existence of divergent ESG ratings can lead to information confusion, making it difficult for managers to determine whether low ratings are caused by poor ESG performance of the enterprise’s production and operation activities or by inconsistencies in the rating agencies’ philosophies or measurement methodologies, which in turn makes it impossible for managers to formulate appropriate countermeasures, reduces their motivation to improve the enterprise’s ESG performance, and is not conducive to achieving good environmental performance through sustainable decision-making processes in business [25,26]. Some studies also mention that in the case of information asymmetry between stakeholders and firms, corporate management may use ESG ratings as a tool for corporate greenwashing [3,7]. These factors are detrimental to the ability of businesses to achieve good environmental performance and support the carbon peaking and carbon neutrality goals through sustainable decision-making processes. In terms of external performance, when different organizations disagree on the ESG ratings of the same listed company, it can reduce the trust of investors, the public, the government and other stakeholders and may also make stakeholders question its sustainability and solvency, thus threatening the legitimacy of the ESG ratings and the green reputation of the company [11,14,26]. Investors reduce their ESG investments and decrease their engagement in corporate ESG issues, which may increase the cost of environmental stewardship for corporations, leading to an increase in carbon emissions [13,27]. After the information provided is analyzed, a hypothesis is proposed:

H1: ESG rating divergence can increase manufacturing firms’ carbon emission intensity.

### The mediating role of green innovation

The term “green innovation” refers to the creation of new goods and services with an emphasis on reducing environmental impact, conserving energy, recycling more, and making better use of existing resources [28]. There are now major environmental concerns as a result of China’s rapid economic expansion. As a result, green innovation has emerged as a viable solution to create a fair balance between economic expansion and environmental preservation. On the one hand, the survival of firms is regarded as the definitive measure of performance; only when green technological innovation enhances firm performance and subsequently increases the viability of green innovative firms can it serve as a sustained catalyst for improved environmental performance. In conjunction with the emergence of green philosophy, an increasing number of companies are being assessed by ESG rating groups [22], and socially responsible enterprises are more inclined to foster green innovation to mitigate environmental pollution [28]. Furthermore, in the context of signaling theory, robust ESG ratings convey to the general public and stakeholders the company’s commitment to environmental accountability and its willingness to allocate resources to sustainable technologies [15]. However, ESG rating divergence exacerbates investor uncertainty regarding a company’s sustainability, potentially prompting companies to adopt strategic behaviors such as prioritizing the quantity of green innovations over quality, thereby conveying misleadingly positive signals to the market about their environmental stewardship and social responsibility, ultimately aiming to maintain their green reputations and mitigate stakeholder mistrust [7]. On the other hand, green innovation is characterized by substantial initial investments, extended payback periods, and uncertain dangers [29]. Furthermore, green patent citations serve as a significant indicator of the “quality” of corporate green innovation, exhibiting a prolonged time lag impact. Certain ESG rating organizations, however, evaluate an organization’s innovative endeavors just within the current year, disregarding temporal lag, resulting in heightened differences in ESG ratings and increased carbon intensity among corporations. After the information provided is analyzed, a hypothesis is proposed:

H2: Green innovation mediates the relationship between emission intensity and ESG rating divergence.

### The moderating role of financing constraints

Financing constraints are the restrictions that companies encounter in terms of funding their intended investments as anticipated. The limitations might stem from various issues, including credit constraints, dependence on bank loans, inability to borrow or issue equity [30], etc. For every business to thrive and last, access to capital is crucial. Insufficient cash can have severe consequences for the financial well-being of an organization, potentially leading to a financial crisis [31]. Financing constraints pose a significant challenge for firms that experience information mismatch [32]. This hinders their ability to allocate resources efficiently and increases their exposure to risk. Research has clearly demonstrated a correlation between corporate governance endorsement and the social responsibility performance of polluting industry firms [18]. Additionally, it has been observed that environmental protection is of paramount importance in a firm’s ability to meet its debt obligations [32]. Furthermore, companies that exhibit greater transparency in ESG practices tend to enjoy lower costs of debt financing [33]. When firms encounter elevated funding hurdles, they often forgo environmental advantages and allocate resources to productive projects characterized by minimal investment, short duration, and rapid returns [34], thus hindering carbon emission reduction efforts. Consequently, the lack of transparency resulting from inconsistent ESG ratings leads enterprises to diminish the quality of their ESG disclosures when confronted with elevated funding requirements, thereby also resulting in a lack of supplementary financial assistance, which ultimately exacerbates their carbon emissions [18,35,36]. After the information provided is analyzed, a hypothesis is proposed:

H3: The greater the financing constraints faced by manufacturing firms are, the stronger the effect of their ESG rating divergence on increasing carbon emission intensity.

### The moderating role of digital transformation

Enterprise digital transformation is the systematic transformation of an enterprise’s organizational structure, business model and production model through the integration of digital technology to achieve transformative upgrading of the operation mode, decision-making system and strategic layout [15,27,37]. Enterprise digital transformation primarily manifests its moderating effect in two key aspects. In terms of direct impact, the long-term solidified business model has led to a series of problems, including low technological content, environmental pollution and lack of market competitiveness [38]. However, digital transformation helps companies develop a more open, innovative and shared strategic concept [1], which leads to sustainable development. Divergent ESG ratings add complexity and uncertainty to investment decisions, making it difficult for investors to assess corporate sustainability efforts [39]. The digital transformation of manufacturing companies can improve transparency and information credibility, effectively reducing the degree of information asymmetry while releasing positive signals to investors [4,40]. It has also been established that digital transformation can significantly improve firms’ carbon disclosure [41] and carbon performance [42] and reduce their carbon emissions [1,43]. In addition, companies applying digital technology to monitor their natural and social environments can also reduce carbon emissions, further exemplifying ESG investment [4,44].

In terms of indirect impacts, the first effect of divergent ESG ratings is to hinder the development of technological innovation, but studies have shown that improved corporate green innovation capacity can mitigate this effect [26,39]. Digital transformation can help improve green innovation and provide new opportunities for companies to achieve rapid development and green transformation. First, digital transformation can facilitate the flow of innovation factors such as knowledge, information and technology among enterprises, and the flow of these key factors is an important means for enterprises to increase the potential of green technology innovation [10]. Second, digital transformation encourages enterprises to move away from the traditional development model, fully optimize all aspects of enterprise production, promote the optimization and adjustment of the structure of production factors, and accurately manage the entire product life cycle, thus promoting green transformation [4,27]. Finally, digital transformation can further enhance green innovation through digital applications such as carbon trading, carbon capture, carbon utilization and carbon sequestration. The enhancement of enterprises’ green technology innovation capacity will accelerate the generation and updating of enterprises’ green technology achievements and greatly improve their production efficiency and resource utilization efficiency, thus reducing their carbon emissions [1]. After the information provided is analyzed, a hypothesis is proposed:

H4: The more digitally transformed a manufacturing company is, the weaker the impact of its ESG rating divergence on increasing carbon intensity.

H5: The digital transformation of enterprises can moderate the indirect impact path of “ESG performance→green innovation→carbon emission intensity”.

## Research design

### Sample selection and data sources

**Fig 1:**
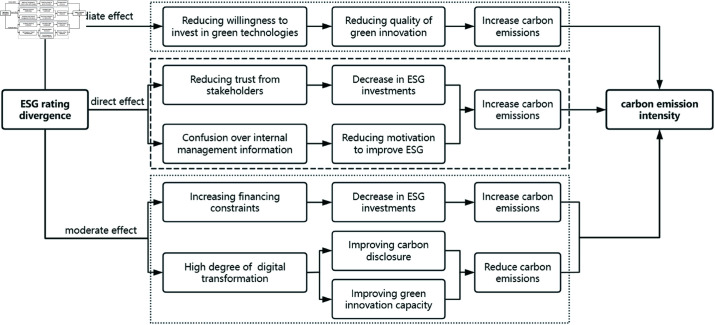
Schematic diagram of the intrinsic mechanism by which ESG rating divergence affects the carbon emission intensity of manufacturing firms.

The present study empirically investigates the influence of ESG rating divergence on the carbon intensity of manufacturing firms listed on the A-share market. The analysis is based on monthly data from 2011–2022. Data sources: Basic corporate information and financial indicators from the CSMAR database, ESG rating information sourced from the official websites of ESG rating agencies, corporate green innovation patent information from the CNRDS database, and carbon intensity-related data from the China Energy Statistical Yearbook and China Statistical Yearbook. The samples underwent additional processing following the procedures outlined in Table 1, yielding valid samples for analysis. The generated data were then processed and analyzed via software tools, including Stata16 and Excel. The specific intrinsic schematic diagram is shown in Fig 1.

**Table 1:** Specific steps.

Steps	Concrete content
(1)	Excluding firms with missing key analytical variables.
(2)	Exclusion of enterprises in the ST or *ST category.
(3)	Excluding firms with significant changes or transformed their primary enterprise throughout the course of the research.
(4)	Mitigating the effects of extreme values by shrinking the tails of continuous variables by 1 percent above and below.

### Variable description

#### Explained variable: Carbon emission intensity.

With reference to Lyu *et al*. [2] and Zhou and Liu [44], this paper quantifies the carbon intensity of manufacturing firms by using the ratio of enterprise CO2 emissions to enterprise operating income. The rate is calculated via estimates of the energy requirements of the industry, using a conversion factor of 2.493 per kilogram of conventional coal carbon dioxide. The calculation formula is as follows:

$$Enterprise\;CO2\;emissions=\;\frac{Enterprise\;operating\;costs\times Total\;industry\;energy\;consumption\times CO2\;conversion\;factors}{Industry\;operating\;costs}$$ (1)

$$Enterprise\;carbon\;emissions\;intensity=\frac{Enterprise\;CO2\;emissions\times 10^{8}}{Enterprise\;operating\;income}$$ (2)

#### Explanatory variable: ESG rating divergence.

With reference to Zhu *et al*. [10] and Avramov *et al*. [13], the rating scores from six agencies, including Bloomberg, Wind, FTSE Russell, China Securities, Syndao and Menglang, for the same firm were utilized as source data. The specific methodology is as follows: (1) Data processing. The ESG ratings of the above rating agencies were harmonized into a standardized format to quantify the level of consistency among the assessment agencies. (2) Sorting process. The ESG ratings of companies assessed by each rating agency are ranked on an annual basis, with the rankings increasing as the score increases. (3) Normalization. The range normalization methodology to normalize firm rankings across rating agencies and ensure that all ESG ratings are between 0 and 1. (4) Formation variables. The standard deviation of the rankings provided by each of the two rating agencies for each firm in a given year is the “difference in paired ratings”. This is done by calculating the inverse of all paired rating differences and taking the logarithm of it as the ESG rating disagreement (ESG_dis) of the firm for the year.

#### Mediating variable: Green innovation.

With reference to Zhu *et al*. [10], the assessment of a firm’s green innovation quality is performed by calculating the total number of citations for green patents filed by the company in the current year, excluding the number of citations by the company itself, and then +1 to calculate the natural logarithm of the citation statistics.

#### Moderator variables: Financial constraint and digital transformation.

Financial constraints: With reference to Hadlock and Pierce [45], this study uses the SA index to quantify the financial limitations faced by manufacturing companies. The index comprises two factors: firm size and firm age. When determining the age of a firm, one must deduct the year of output from the year of establishment, as shown below.

$$SA=-0.737\times Size+0.043\times {Size}^{2}-0.04\times Age$$ (3)

Digital transformation: With reference to Wu *et al*. [46], find the overall frequency of the terms used in the annual report to describe digital technology, cloud computing, blockchain, big data, and artificial intelligence. Then, add 1 is added to the result, and the natural logarithm is applied to create a metric for the enterprise’s digital transformation.

#### Control variables.

With reference to Cai *et al*. [5], Li and Huang [47] and Franca *et al*. [48], Table 2 show the control variables used for this study, which were selected on the basis of references to relevant domestic and international research.

**Table 2:** Variable definitions.

Variable Type	Variable Name	Variable Symbol	Variable Definition
Explainedvariables	Carbon emission intensity	CEI	Enterprise CO2 emissions×10^8^/Enterprise operating income
Explanatoryvariable	ESG rating divergence	ESG_dis	Ln(1/Standard deviation of ESG ratings from various rating agencies)
	Green innovation	GI	Ln (Sum of citations in the next two years for green patents filed by the enterprise in the current year - The number of citations by the enterprise itself + 1)
Moderatorvariable	Financial constraint	FC	SA index
	Digital transformation	DIG	Ln(Total number of times AI technology, cloud computing technology, blockchain technology, big data technology, and digital technology applications appear as keywords in the annual report +1)
Controlvariables	Natural logarithm of firm age	lage	Ln(Sample year -Year of establishment+1)
	Growth	growth	(Operating income for the current year - Operating income for the previous year)/Previous year’s operating income
	Leverage ratio	lev	Ratio of total liabilities to total assets at the end of the period
	Nature of equity	state	State-owned enterprises are assigned a value of 1 and non-state-owned enterprises are assigned a value of 0.
	Asset structure	as	Ratio of current assets to total assets at the end of the period
	Shareholding ratio of the largest shareholder	top1	Number of shares held by the largest shareholder/Total number of shares
	Tobin’s Q-value	tobin’ q	Market capitalization/Total assets

### Model setting

#### Baseline model.

These steps are used to construct the basic regression model used in this study to confirm that ESG rating divergence affects the carbon emission intensity of manufacturing enterprises:

$${CEI}_{it}=\alpha_{0}+\alpha_{1}{ESG\_dis}_{it}+\alpha_{2}{Control}_{it}+\varphi_{t}+\eta_{j}+\mu_{k}+\varepsilon_{it}$$ (4)

where *CEIit* represents the carbon emission intensity of firm i in year t, ${ESG\_dis}_{it}$ is the ESG rating divergence of firm i in year t, *Control*_*it*_ is the corresponding control variable, and $\varepsilon_{it}$ is a random disturbance term. $\varphi_{t}$, $\eta_{j}$ and $\mu_{k}$ are year, industry, and province fixed effects to control for changes in macroeconomic trends, industries, and cities that may be brought about each year. If the coefficient $\alpha_{1}$ is negatively significant, H1 is verified.

#### Mediation effects model.

With reference to Wen and Ye [49] and Baron and Kenny [50], using green innovation as a mediator, a mediation effect model was developed to investigate the mechanism of ESG_dis on CEI in manufacturing companies as follows:

$${GI}_{it}=\beta_{0}+\beta_{1}{ESG\_dis}_{it}+\beta_{2}{Control}_{it}+\varphi_{t}+\eta_{j}+\mu_{k}+\varepsilon_{it}$$ (5)

$${CEI}_{it}=\gamma_{0}+\gamma_{1}{ESG\_dis}_{it}+\gamma_{2}{GI}_{it}+\gamma_{3}{Control}_{it}+\varphi_{t}+\eta_{j}+\mu_{k}+\varepsilon_{it}$$ (6)

where, *GI*_*it*_ represents the mediating variable of green innovation; if $\beta_{1}$ is significant and $\gamma_{2}$ is significant, then it suggests the existence of mediation, and if $\gamma_{1}$ is significant at the same time, then H2 holds.

#### Moderated effects model.

In an effort to evaluate the moderating influence of financing constraints and the degree of digital transformation of enterprises, a specific model is constructed as follows with reference to Wen and Ye [51]:

$${CEI}_{it}=b_{0}+b_{1}{ESG\_dis}_{it}+b_{2}W_{it}+b_{3}{ESG\_dis}_{it}\times W_{it}+b_{4}{Control}_{it}+\varphi_{t}+\eta_{j}+\mu_{k}+\varepsilon_{it}$$ (7)

$${GI}_{it}=c_{0}+c_{1}{ESG\_dis}_{it}+c_{2}W_{it}+c_{3}{ESG\_dis}_{it}\times W_{it}+c_{4}{Control}_{it}+\varphi_{t}+\eta_{j}+\mu_{k}+\varepsilon_{it}$$ (8)

$${CEI}_{it}=\lambda_{0}+\lambda_{1}{ESG\_dis}_{it}+\lambda_{2}W_{it}+\lambda_{3}{ESG\_dis}_{it}\times W_{it}+\lambda_{4}{GI}_{it}+\lambda_{5}{GI}_{it}\times W_{it}\;\;+\lambda_{6}{Control}_{it}+\varphi_{t}+\eta_{j}+\mu_{k}+\varepsilon_{it}$$ (9)

where *W*_*it*_ represents the moderating variable financing constraints or firm digital transformation. If *b*_3_ is significant, financing constraints can moderate the main effect, hypothesis H3 and H4a holds. If *c*_3_ and $\lambda_{4}$ are significant, this indicates that the moderating variable can moderate the first half of the path (ESG_dis→GI). If *c*_1_ and $\lambda_{5}$ are significant, this indicates that the moderating variable can moderate the second half of the path (GI→CEI). If *c*_3_ and $\lambda_{5}$ are significant, this indicates that the moderating variable can moderate the first and second half segment paths, and hypothesis H4b holds.

## Empirical analyses

### Descriptive statistics

Table 3 below presents the study’s variable descriptive statistics. From 2011 to 2022, manufacturing firms had an average carbon intensity of 0.441, with a dispersion of 0.569. The values inside this range for carbon emission intensity during this period were between 0.008 and 3.380. The average ESG rating divergence is 2.648, with a dispersion of 0.784. The values inside this range for ESG rating divergence during this period were between 0 and 5.829, suggesting significant variation in CEI and ESG_dis among manufacturing firms. All other variables exhibited varying disparities, with the Top 1 difference being particularly pronounced (SD=14.061). To ensure that issues with multiple covariances do not arise, this study conducted a variance inflation factor (VIF) test for each variable. With reference to Zhou *et al*. [26], the results indicate that the VIF of each variable falls between 1 and 3, suggesting that there is no significant issue of multiple covariance. Therefore, the sample data can be further analyzed through regression.

**Table 3:** Results of descriptive statistics.

VarName	Obs	Mean	SD	Min	Median	Max	VIF
*CEI*	13226	0.441	0.569	0.008	0.144	3.380	
ESG_dis	13226	2.648	0.784	1.016	2.501	9.643	1.09
*GI*	13226	0.920	1.323	0.000	0.000	5.829	1.45
*SA*	13226	-74.965	1.486	-77.629	-75.207	-70.519	1.98
*DIG*	13226	1.417	1.266	0.000	1.099	4.898	1.13
*lage*	13226	2.983	0.285	1.609	2.996	3.611	1.13
*growth*	13226	0.129	0.211	-0.325	0.087	2.187	1.10
*lev*	13226	0.400	0.185	0.035	0.396	0.924	1.36
*state*	13226	0.290	0.454	0.000	0.000	1.000	1.27
*as*	13226	0.579	0.165	0.084	0.587	0.942	1.14
*top*1	13226	33.609	14.061	3.620	31.310	75.000	1.08
$tobin^{\prime }q$	13226	2.068	1.327	0.821	1.644	10.282	1.15

### Benchmark regression analysis results

A review of ESG rating divergence regression findings on the carbon intensity of manufacturing firms is shown in Table 4. In reference to Yu *et al*. [34], based solely on the fixed effects of year, industry, and province, column (1) displays the regression findings free of control variables. Column (2) shows the regression results with control variables but considering only year fixed effects. Column (3) shows the regression results with industry fixed effects added to column (2), and column (4) shows the regression results with province fixed effects added to column (3). Furthermore, in line with Aouadi and Marsat [52], this study utilizes company-level clustering standard errors in all estimations. The findings from all three studies demonstrate a clear and statistically significant negative impact of ESG rating divergence on the CEI at the 1% level of significance. Specifically, the regression coefficient in Column (3) is 0.012, suggesting that a 1-unit increase in the annual average value of ESG performance for manufacturing enterprises increases their carbon intensity by 1.2%. Consistent with the theoretical study of H1, research suggests that ESG rating divergence increases the carbon intensity of manufacturing firms.

**Table 4:** Regression results for ESG rating divergence and carbon emission intensity.

	(1)	(2)	(3)	(4)
**CEI**	**CEI**	**CEI**	**CEI**
ESG_dis	0.010 * * *	0.038 * * *	0.012 * * *	0.012 * * *
	(3.60)	(4.10)	(4.46)	(4.50)
*lage*		0.034	0.015 *	0.014
		(0.86)	(1.72)	(1.59)
*growth*		0.032	-0.051 * * *	-0.053 * * *
		(1.09)	(-6.19)	(-6.35)
*lev*		0.055	0.134 * * *	0.136 * * *
		(0.94)	(9.16)	(9.09)
*state*		0.139 * *	0.016 * * *	0.019 * * *
		(5.02)	(2.59)	(2.86)
*as*		-0.999 ***	0.029***	0.028*
		(-14.73)	(1.76)	(1.69)
*top*1		0.001	0.000	0.000
		(1.60)	(0.94)	(1.03)
$tobin^{\prime }q$		-0.038***	-0.014***	-0.014***
		(-6.30)	(-7.48)	(-7.37)
_cons	0.415***	0.785***	0.318***	0.320***
	(51.97)	(5.64)	(10.21)	(10.01)
*Year*	YES	YES	YES	YES
*Industry*	YES	NO	YES	YES
*Area*	YES	NO	NO	YES
*N*	13226	13226	13226	13226
*R*2	0.923	0.123	0.926	0.928

***denotes p<0.01, **denotes p<0.05, *denotes p<0.1, and values in parentheses are t values adjusted for firm-level clustering, the identical thing underneath.

### Robustness tests

#### Extended viewing window.

The objective of this investigation is to investigate the pre-post correlation between the CEI of manufacturing enterprises and their ESG_dis. To accomplish this, the approach used in this study is based on the work of Zhang and Han [53]. The explanatory variable (ESG_dis) is lagged by 1–3 periods, whereas the explanatory variable (CEI) is front-loaded by 1–3 periods. The findings of the regression analysis can be found at Table 5. The study reveals that ESG rating divergence has a strong and statistically significant negative effect on CEI. This effect holds true in both the lagged model (Columns (1)-(3)) and the prior model (Columns (4)-(6)) and is not influenced by time-restricted recessions. Further evidence for Hypothesis H1 is provided by this discovery, which demonstrates that manufacturing firms’ carbon intensity is significantly affected by ESG rating divergence.

**Table 5:** Extending the observation window.

	(1)	(2)	(3)	(4)	(5)	(6)	(7)
	CEI	CEI	CEI	F.CEI	F2.CEI	F3.CEI	CEI1
L.ESG_dis	0.011***						
	(3.66)						
L2.ESG_dis		0.013***					
		(3.85)					
L3.ESG_dis			0.016***				
			(4.43)				
ESG_dis				0.010***	0.012***	0.019***	0.007***
				(3.47)	(3.37)	(4.18)	(3.16)
_cons	0.326***	0.320***	0.307***	0.323***	0.292***	0.275***	0.253***
	(9.12)	(8.03)	(6.67)	(9.25)	(7.34)	(6.03)	(11.37)
*Year*/*Industry*/*Area*	YES	YES	YES	YES	YES	YES	YES
*Control*	YES	YES	YES	YES	YES	YES	YES
N	10076	7684	5754	10076	7684	5754	13226
*R*2	0.929	0.929	0.929	0.914	0.903	0.890	0.968

#### Replacement of explanatory variables.

To further verify the robustness of H1, this study refers to Wang *et al*. [54] to measure the carbon emissions of the manufacturing industry according to the CO2 emission estimation formula provided by IPCC (2006), which is shown below. In the formula, i denotes nine major energy sources: raw coal, coke, crude oil, gasoline, kerosene, diesel fuel, fuel oil, natural gas and electricity; *K*_*i*_ is the carbon emission factor of the ith energy source (10,000 tons/million tons of standard coal); and *E*_*i*_ is the consumption of the ith energy source (measured by 10,000 tons of standard coal). Referring again to Wang *et al*. [42] and Cao *et al*. [55], the recalculated CO2 emissions from the manufacturing sector are brought into Equation (1) and Equation (2) to obtain CEI1, and the regression results are shown in Column (7) in Table 5. CEI1 is positively significant for ESG_dis at the 1% level, which is consistent with the baseline regression results, again validating H1.

$$Industry\;carbon\;emission=\frac{44}{12}\sum_{i=1}^{9}K_{i}E_{i}$$ (10)

### Heterogeneity analyses

#### Instrumental variable 2SLS test.

To address the potential relationship between ESG_dis and CEI, two instrumental variables are created. The second instrumental variable (IV1) is derived from the average ESG performance of the same sector and province during the same year, as studied by Chen *et al*. [56]. From a correlational standpoint, the ESG performance of individual firms can exert an economic influence on the entire industry through information spillovers. As a result, there may be a strong correlation between industry ESG rating divergence and enterprise-level ESG rating divergence [10]. From the standpoint of exogeneity, the ESG_dis of other firms within the same industry and province during the same year will not exert a direct influence on their carbon emissions. The model is evaluated via 2SLS, and the instrumental variable estimates are presented in Table 6. Columns (1) and (2) present the outcomes of the first- and second-stage regressions for IV1. The regression results indicate a strong and favorable relationship between IV1 and ESG_dis, the Cragg–Donald Wald F statistic (IV1=659.977) and the Kleibergen–Paap rk Wald F statistic (IV1=219.536) exceed the crucial figure of 16.38 by a significant margin of 10%, thus resolving the problem of ineffective instrumental variables. For the nonidentifiable test display, Kleibergenâ€’Paap mk LM has a value of 122.273 (IV1, P=0.000). It is clear from the considerable rejection of the initial, unidentified hypothesis that IV1 has a strong correlation with endogenous factors. The findings in columns (1) and (2) show that even with bidirectional causality mitigated, ESG_dis can have a substantial effect on manufacturing enterprises’ CEI.

**Table 6:** Endogeneity test.

	(1)	(2)	(3)	(4)
	ESG_dis	CEI	dum_ESG_dis	CEI
*IV*1	0.368***			
	(14.82)			
*IV*2			0.370***	
			(3.37)	
ESG_dis		0.0464***		0.013***
		(3.27)		(4.55)
*IMR*				-0.181*
				(-1.77)
*Year*/*Industry*/*Area*	YES	YES		YES
*Control*	YES	YES		YES
*N*	13226	13226	9018	9141
Kleibergen−PaaprkLMstatistic	122.273eak [0.000]			
Cragg−DonaldWaldFstatistic		659.977		
Kleibergen−PaaprkWaldFstatistic		219.536		
10%maximalIVsize		16.38		

[ ] is the p-value of the statistic.

#### Heckman two-step method.

This study opted to utilize the Heckman two-step method to ensure that the samples were not biased. The second instrumental variable (IV2) is based on the lagged first order of ESG rating divergence, as identified by Li *et al*. [57]. From the standpoint of instrumental variable correlation and exogeneity, the lagged first order of ESG_dis encompasses solely firm-specific information, encouraging firms to prioritize their ESG management; however, it does not directly influence firms’ carbon emission intensity [56,57]. In accordance with the study by Zhang and Han [53], a dummy variable for ESG_dis (dum_ESG_dis) is initially created to indicate the presence or absence of divergence in ESG ratings. The dummy variable is assigned a value of 1 when there is a discrepancy in ESG ratings among manufacturing enterprises and 0 otherwise. Additionally, the Inverse Mills Ratio was computed via the probit model following the incorporation of year, industry, area, and control variables. Ultimately, the IMR is subsequently incorporated into the second-stage model within the regression analysis. The findings, as displayed in columns (3) and (4) of Table 6, indicate that the regression coefficient for IV2 was 0.370 (p<0.01). After adding IMR, the IMR coefficient attains significance at the 10% level, signifying a substantial issue of sample selection bias. That is, controlling for the potential effect of sample selection bias, the coefficient of ESG_dis remains positively significant at the 1% level, indicating that ESG_dis still promotes robust conclusions on the carbon emissions of manufacturing firms.

### Impact mechanism tests

#### Mediating effect of GI.

Both the direct and indirect effects of ESG_dis on manufacturing companies’ CEI are statistically noteworthy, as shown in Table 9 columns (1) and (2), respectively, of . In column (1), the ESG_dis coefficient is –0.189, suggesting a correlation at the 1% significance level. Moving on to column (2), the GI coefficient is -0.008, which is also at the 1% level. Furthermore, in column (2), ESG_dis has a coefficient of 0.010, which is statistically significant at the 1% level. The study suggests that GI has a limited role in mediating the relationship between ESG_dis and CEI. ESG_dis adversely affects the CEI governance of manufacturing enterprises by inhibiting their GI. To learn more about how GI acts as a mediator, this paper used the bootstrap technique, running 500 sample iterations. The results, with a 95% confidence interval of [0.0009, 0.0018], confirm the validity of the study’s results because the interval is not empty. As a result, A-share listed manufacturing firms form a path of “ESG rating divergence → (reduce) green innovation → (increase) carbon emission intensity”, assuming that H2 holds.

#### Moderating effect of SA.

Financial support is essential for promoting corporate carbon emission reduction. For instance, financial institutions prioritize companies with strong ESG performance when developing credit policies. They encourage businesses to cut their carbon emissions by offering advantageous loan terms and helping to coordinate the flow of capital and technological assets into concentrated areas of resource availability [22]. Columns (3), (4), and (5) of Table 7 present the findings of the regression analyzing the impact of funding limitations on ESG rating divergence, including both direct and indirect effects. The data in column (3) show that the regression coefficients of ESG_dis × SA are significantly positively correlated at the 1% level, indicating that financing constraints strengthen the effect of ESG rating divergence on the promotion of carbon emissions. If enterprises have significant funding limitations, disparities in ESG ratings exacerbate carbon emissions, contingent upon the validity of H3. The coefficients of ESG_dis and ESG_dis × SA in column (4) are significant, but the coefficients of GI and GI × SA in column (5) are not significant; thus, hypothesis H3 is tested again.

**Table 7:** Testing of mediating and moderating effects.

	(1) GI	(2) CEI	(3) CEI	(4) GI	(5) CEI	(6) CEI	(7) GI	(8) CEI
ESG_dis	-0.189***	0.010***	0.482***	-1.702*	0.440**	0.016***	-0.098***	0.014***
	(-10.56)	(3.93)	(2.65)	(-1.81)	(2.39)	(3.64)	(-4.07)	(3.22)
*GI*		-0.008***			-0.137			-0.012***
		(-2.65)			(-0.87)			(-2.68)
*SA*			-0.029***	0.512***	-0.024***			
			(-4.24)	(13.38)	(-3.22)			
ESG_dis×SA			0.006***	-0.022*	0.006**			
			(2.62)	(-1.76)	(2.36)			
GI×SA					-0.002			
					(-0.87)			
*DIG*						0.002	0.341***	-0.001
						(0.38)	(7.62)	(-0.11)
ESG_dis×DIG						-0.003*	-0.055***	-0.002
						(-1.73)	(-4.00)	(-1.43)
GI×DIG								0.003**
								(2.09)
_cons	0.274	0.322***	-1.896***	40.139***	-1.544***	0.317***	-0.181	0.325***
	(0.95)	(10.18)	(-3.62)	(13.70)	(-2.68)	(9.29)	(-0.62)	(9.48)
*Year*/*Industry*/*Area*	YES	YES	YES	YES	YES	YES	YES	YES
*Control*	YES	YES	YES	YES	YES	YES	YES	YES
*N*	13226	13226	13226	13226	13226	13226	13226	13226
*R*2	0.283	0.928	0.928	0.424	0.928	0.928	0.309	0.928

#### Moderating effect of DIG.

Using green innovation as a lens, this research looks at the direct and indirect implications of digital transformation in enterprises. It is possible to access the regression results in columns (6), (7), and (8) of Table 7, where the coefficient of column (6) ESG_dis × DIG is significant at the 10% level, indicating that the degree of digital transformation of the firm moderates the main effect. This finding indicates that a firm’s digital transformation can regulate the immediate impact of ESG rating divergence on carbon intensity; specifically, a high level of digital transformation allows ESG rating divergence to alleviate the carbon emissions of manufacturing enterprises. Hypothesis H4 is thus verified. The coefficients of columns (7) ESG_dis × DIG and (8) GI are both significant at the 1% level, indicating that firms’ digital transformation moderates the first half of the segment (ESG_dis→GI). The coefficients of column (7) ESG_dis and column (8) GI×DIG are both significant at the 5% level, indicating that firms’ digital transformation moderates the second half of the segment (GI→CEI). Because the coefficient of ESG_dis×DIG in the first half is -0.055 and the coefficient of GI×DIG is 0.003, the digital transformation of enterprises weakens the inhibitory effect of ESG rating divergences on green innovations and promotes the inhibitory effect of green innovations on the intensity of carbon emissions, thus verifying hypothesis H5.

### Heterogeneity analyses

#### Heterogeneity of property rights.

In contrast, listed firms with varying ownership structures have distinct motivations for disclosing their ESG practices. Nonstate-owned listed companies (NSOEs), being primarily market participants, are focused primarily on achieving higher economic returns. State-owned listed companies (SOEs) prioritize institutional factors, policy factors, and social reactions, which stands in stark contrast to other entities. Furthermore, SOEs face greater social pressure and societal expectations than do NSOEs [58]. Alternatively, SOEs and NSOEs have distinct ESG disclosure priorities. SOEs prioritize national policies and implement ESG practices aligned with the country’s development goals. Nevertheless, NSOEs prioritize the needs of stakeholders [18]. Thus, this research builds on previous work by Zhou *et al* [58]. To analyze how ESG_dis factors affect carbon emissions from manufacturing companies with different types of property rights, values are assigned to property rights attributes (1=state-owned firms, 0=nonstate-owned firms). The results are presented in columns (1) and (2) of Table 8. The CEI of ESG_dis for both SOEs and NSOEs is statistically significant at the 1% level. However, SOEs have greater absolute values for the ESG regression coefficients than do NSOEs. This provides more evidence that the ESG_dis of manufacturing businesses, whether owned by the state or not, has a substantial role in managing carbon emissions. The effect of ESG_dis on carbon emissions is more pronounced for SOEs than for NSOEs in manufacturing.

**Table 8:** Heterogeneous effects of property rights and areas.

	(1)	(2)	(3)	(4)	(5)
	State-owned firms	Non-state-owned firms	Eastern area	Central area	Western area
ESG_dis	0.023***	0.007***	0.010***	0.015*	0.023**
	(3.27)	(2.84)	(3.44)	(1.78)	(2.54)
_cons	0.327***	0.312***	0.298***	0.366***	0.433***
	(3.91)	(9.27)	(8.58)	(3.60)	(3.95)
*Year*/*Industry*/*Area*	YES	YES	YES	YES	YES
*Control*	YES	YES	YES	YES	YES
*N*	3839	9387	9039	1539	2083
*R*2	0.911	0.941	0.934	0.935	0.908

#### Heterogeneity of areas.

Varying levels of regional development, economic status, and industrial composition can result in geographical disparities in the magnitude of enterprises’ carbon emission intensity [1]. This work utilizes Chen *et al*. [1] to assign values to areas (1=Eastern area, 2=Central area, 3=Western area) to confirm how ESG_dis affects the CEI of different kinds of manufacturing companies. The results shown in rows (3) through (5) of Table 8 reveal that the ESG factors of manufacturing companies in regions to the east, center, and west have a positive effect on CEI, which is statistically significant at the 1%, 5% and 10% levels. ESG_dis is most pronounced in the eastern region, whereas the regression coefficient of ESG_dis is greatest in the western region, indicating that the ESG_dis of manufacturing businesses in the western region exerts the most substantial positive influence on carbon emission intensity.

#### Heterogeneity of industries.

Largely environmentally harmful sectors use greater amounts of energy in their manufacturing operations and often face more financial constraints to adopt energy-saving technologies and reduce carbon emissions [59]. Confirming the effect of ESG_dis on the CEI of different kinds of manufacturing companies is the aim; with reference to Wang and Yang [6], the sample of manufacturing firms with CSRC industry codes C15, C17, C18, C19, C22, C25, C26, C27, C28, C29, C31, and C32 are classified as heavily polluting industries (given a value of 1) and nonpolluting industries (given a value of 0). The outcomes are displayed in Table 9, namely, in columns (1) and (2). The ESG disclosure of manufacturing firms in heavily polluting industries positively and significantly influences the CEI at the 1% level, whereas it is not significant in nonheavily polluting industries, indicating that the ESG disclosure of firms in heavily polluting sectors substantially contributes to their CEI.

**Table 9:** Heterogeneous effects of heavily polluting industries and degree of market competition.

	(1)	(2)	(3)	(4)
	Heavy-polluting industries	Non-heavy-polluting industries	High market competition	Low market competition
ESG_dis	0.029	0.002***	0.009***	0.012***
	(4.71)	(1.57)	(3.82)	(2.74)
_cons	0.455 ***	0.223 ***	0.299 ***	0.420 ***
	(5.59)	(11.61)	(9.57)	(8.78)
*Year*/*Industry*/*Area*	YES	YES	YES	YES
*Control*	YES	YES	YES	YES
*N*	5070	8156	7031	6195
*R*2	0.902	0.941	0.946	0.928

#### Degree of market competition.

Signaling theory suggests that when market competition is intense, it can intensify adversarial relationships between firms and create more pressure related to ESG_dis. In highly competitive markets, competitors are motivated to showcase their strengths in different ways. Companies with excellent ESG ratings seize the opportunity to enhance their growth and governance dynamics [22]. In this study, we look at manufacturing firms operating at different levels of market competitiveness to determine how ESG_dis affects their CEI. Using Lerner’s index, as proposed by Li *et al*. [57], we may quantify the level of market rivalry. Two groups are formed from the sample according to the median of Lerner’s index (high degree of market competition=1, low degree of market competition=0). Below, in Table 9, the results are presented in columns (3) and (4). At the 1% level of significance, the analysis shows that manufacturing businesses’ ESG_dis positively affects CEI, irrespective of the degree of market rivalry. Moreover, the regression coefficients of ESG_dis for low market competition are greater than those for high market competition, suggesting that industrial enterprises operating in low competitive markets are more strongly affected by the ability of ESG_dis to increase carbon emissions. Therefore, the impact of ESG_dis on carbon emissions is more significant for low-market-competitive manufacturing enterprises than for high-market-competitive firms.

## Conclusions and policy recommendations

### Conclusions

As carbon emission reduction has reached a global consensus, countries worldwide are actively promoting the development of green and minimal carbon emissions. As the foremost contributor to global carbon emissions, China must prioritize sustainable low-carbon development if it wants to maintain its current rate of economic growth and continue to expand [1,22,60]. Using data from 2011-2022, this study looks at how ESG_dis relates to CEI in Chinese A-share listed industrial companies. The analysis employs high-dimensional fixed effects and yields the following findings: (1) There are two separate ways in which ESG_dis might affect the intensity of carbon emissions. With respect to increasing carbon emissions, ESG_dis is key in manufacturing enterprises through the direct path. After we carefully consider lagged explanatory variables, preexplained variables, replace explanatory variables, construct instrumental variables, and conduct the Heckman two-step method, it is evident that the positive promotion effect remains valid. The second is the mediation path, where increased green innovation mediates the relationship between ESG_dis and CEI. (2) Financing constraints and corporate digital transformation both impact carbon emissions in ESG rating divergences. Increasing financing constraints magnify the impact of ESG rating divergence on carbon intensity, whereas enterprise digital transformation mitigates the impact of ESG rating divergence on carbon intensity. In addition, digital transformation affects the indirect path of “ESG rating divergence → green innovation → carbon emission intensity”, i.e., it negatively moderates the negative impact of ESG rating divergence on green innovation and simultaneously strengthens the inhibitory effect of green innovation on carbon emission intensity. (3) Companies in low competitive markets, those in strongly polluting industries, those located in eastern areas, and those owned by the state have a more noticeable effect on ESG_dis in terms of carbon emissions.

### Research contribution

First, based on the conclusion that ESG ratings can reduce carbon emissions [15–17], which has been confirmed in the literature, this study explores the mechanism of ESG rating divergence on the carbon emission intensity of manufacturing firms from the perspective of ESG rating-derived information, enriching the research on the environmental effects of ESG rating divergence in Chinese manufacturing firms. The results show that reducing the ESG rating divergence of manufacturing firms can effectively reduce carbon emissions, suggesting that the proposed harmonization of ESG rating systems is a contributing factor in achieving sustainable corporate development.

Second, his study reveals mechanisms for internalizing ESG rating divergence into green innovation to drive carbon governance. Unlike previous studies that used the number of green patents to measure the degree of green innovation of an enterprise, this study uses the increase in subsequent green patent citations to represent the green innovation of an enterprise, which better reflects the actual commercial value of an enterprise’s green patents [22]. Our study reveals that firms’ ESG rating divergence can increase carbon emissions by inhibiting green innovation, which is consistent with the findings of existing studies [7,10]. That is, ESG rating divergence limits the pursuit of green innovation by manufacturing firms, thus hindering the advancement of energy conservation and emission reduction in manufacturing firms. However, this finding also suggests that lowering the divergence of ESG ratings of manufacturing firms can contribute to the improvement of green innovation and provide a new source for manufacturing firms to maintain their competitive advantage and reduce carbon emissions.

Third, we find that firms can offset the risk from ESG rating divergence when they have a higher degree of digital transformation, whereas higher financing constraints can act as a signal amplifier for the impact of ESG rating divergence on carbon emissions. On the one hand, relevant studies have shown that enterprise digital transformation not only promotes carbon emission reduction [1] but can also lead to ESG ratings to help new energy enterprises’ green innovation “improve quality and efficiency” [61]. Increasing the degree of digital transformation of firms can mitigate the dampening effect of ESG rating divergence on green innovation, which also suggests that accelerating the digital transformation process of manufacturing firms can help drive their green development. Therefore, this study investigates the moderating role of corporate digital transformation on the direct and indirect effects of “ESG rating divergence - Carbon emission intensity”. On the other hand, research has argued not only that financing constraints can increase the carbon intensity of firms [34]. It also cuts across variables such as managerial myopia, supplier concentration, and green bonds, confirming that easing financing constraints can effectively reduce firms’ carbon emissions [62–64]. Therefore, this study cuts through ESG rating divergence to explore the mechanism of financing constraints in their carbon increase.

Fourth, heterogeneity analysis based on differences in ESG disclosure purpose and focus, regional development, industry pollution level, and signaling. The results suggest that the impact of ESG rating divergence on carbon emission intensity is more pronounced for firms that are state-owned, located in Western China, in heavily polluting industries, and with a low degree of market competition. The reasons for this may be that nonstate-owned firms are more focused on economic returns and stakeholder needs [18,57]; the economic structure of the western region is relatively more traditional, with a larger share of heavy industries and industries with high carbon emissions; and excellent ESG ratings are more likely to stimulate the development and governance dynamics of firms with high degrees of market competitiveness [22].

### Policy recommendations

The results shown above can aid Chinese manufacturing enterprises in their pursuit of sustainable development by assisting them in promoting the optimization of energy use and the mitigation of emissions. This, in turn, can help them accomplish their carbon peaking and carbon neutrality objectives. Accordingly, this study makes the following policy recommendations. First, the government should develop a cohesive ESG rating framework that harmonizes global standards with regional nuances, promotes the ongoing enhancement of ESG evaluation methodologies by third parties, mitigates the adverse effects of rating discrepancies on corporations, and enhances ESG efficacy in regulating corporate carbon emissions. Moreover, improving the ESG disclosure system enables the ESG ratings of enterprises to be clearly presented to the public and investors and promotes the fulfillment of corporate social responsibility and the realization of sustainable development. In addition, it emphasizes investor attention to corporate social responsibility and environmental sustainability, thereby increasing the capital market recognition of companies with outstanding ESG ratings. Second, in the face of the high carbon risk posed by ESG ratings, the government can create a favorable financing environment for enterprises to implement low-carbon development strategies by providing government subsidies or preferential policies, etc., and guide financial resources to support the development of low-carbon enterprises accurately. Companies can also respond proactively by signaling positive actions such as proactive ESG disclosure and other strategies. Finally, the important role of digital transformation in ESG rating divergence has enhanced carbon pathways. The government should accelerate the construction of digital infrastructure and provide information infrastructure, including networks, hardware equipment and software systems, for the digital transformation of enterprises to help them improve the level of green innovation and lead low-carbon development. Enterprises utilize cloud computing and other digital technologies to establish an all-round low-carbon development paradigm from strategic concepts, information disclosure, and innovation capabilities to regulatory systems and effectively improve the carbon management level of enterprises to promote the coordination and unity of their economic, social and ecological benefits.

### Limitations and future research directions

The following caveats are associated with this research. First, this study uses a proxy variable for carbon intensity rather than directly reported emissions data. Limitations in data availability may result in an overestimation or underestimation of each company’s actual emissions; therefore, our findings about ESG_dis and CEI may lack precision compared with their potential accuracy. Second, the research sample for this study consists of manufacturing enterprises listed on China’s A-share market, and the analysis can be expanded to different countries and industries in the future. Finally, although this study considers the mediating variable of green innovation and the moderating variables of financing constraints and firms’ digital transformation, it is possible that these criteria do not provide a complete picture of the relationship. Future research should employ a multifaceted approach to learn more about the effects of ESG_dis on future efforts to reduce carbon emissions.
